# Ad hoc digital communication and assessment during clinical placements in nursing education; a qualitative research study of students’, clinical instructors’, and teachers’ experiences

**DOI:** 10.1371/journal.pone.0287438

**Published:** 2023-07-21

**Authors:** Edel Jannecke Svendsen, Randi Opheim, Bjørg Elisabeth Hermansen, Camilla Hardeland

**Affiliations:** 1 Department of Nursing and Health Promotion, Oslo Metropolitan University, Olso, Norway; 2 Department of Research, Sunnaas Rehabilitation Hospital, Nesoddtangen, Norway; 3 Institute of Health and Society, Faculty of Medicine, University of Oslo, Oslo, Norway; 4 Oslo University Hospital, Oslo, Norway; 5 Department of Health and Social Studies, Østfold University College, Halden, Norway; University of Science and Technology of Fujairah, YEMEN

## Abstract

**Background:**

There was a concern about the shortage of nurses that resulted from the Covid-19 pandemic. Therefore, universities and university colleges were instructed to continue educating nursing professionals but were challenged by the social distancing and the limitations of clinical placements and clinical-field instructors. Clinical placement is essential in the students’ development of practical skills and knowledge. Thus, transitioning to a digital follow-up platform of communication with the students between the universities/college and the clinical practice sites became necessary.

**Purpose:**

To obtain knowledge about the experiences from the university/college teachers, students, and clinical-field instructors regarding the transition to a digital learning environment that resulted from the COVID-19 pandemic.

**Methods:**

Qualitative individual digital interviews were conducted for data collection at three different nursing education programs from three Norwegian university/university college sites. Five students, four clinical-field instructors, and nine university/college teachers participated (n = 18).

**Results:**

The inductive analyses identified two main themes: (1) Efficiency compromising pedagogical quality, and (2) Digital alienation.

**Conclusions:**

Students and university/college teachers were worried about fluctuating quality with digital pedagogical. There were concerns that the students educated during this period will have reduced clinical competencies.

## Introduction

Over the last three decades, many universities and university colleges have used a variety of digital platforms as a possibility for teaching modernization and efficacy. The digitalization of nursing education has typically involved E-learning initiatives and virtual simulations [1], but digitally hosted lectures and evaluation meetings with students in clinical practice were less common pre- pandemic. A review concluded that for an E-learning-based program to be integrated into a curriculum, staff and students require appropriate training [2]. When the pandemic started there was little time for training on how to use digital tools. However, Langegård, Kiani [3] identified how using digital tools in higher education had increased over the last decade. Digitalization has thus introduced a new dimension in teachers’ pedagogical skills and competences, which can be referred to as pedagogical digital competence. This digital competence comprises the ability to plan, to conduct, to evaluate and revise information and communication technology (ICT)-supported teaching, as well as use current research in supporting students’ learning in the best possible way [4], especially due to the physical restrictions with the COVID-19 pandemic. When new communication technology was introduced into nursing education, the university/college teachers reported that it was challenging to teach and to support the students, particularly when they were unfamiliar with aspects of the platforms and software [5].

### Background

Through clinical placements, nursing students acquire practical skills and theoretical knowledge while developing their professionalism in an actual clinical environments [6]. Integrating theory and practice is easier for students to comprehend when strong cooperation between educational and clinical institutions exists [7, 8]. During clinical placements, the students are usually assigned one clinical field-instructor. This instructor is a nurse who provides support, supervision, and evaluation of the student’s learning process, but is less likely to have formal pedagogical education. During the clinical placement, the teachers monitor the evaluation meetings the student and instructor. An Australian survey revealed that resource provision and universities’ communication with clinical-field instructors are challenging [9]. Some clinical-field instructors experience a lack of interest and cooperation from the university/college teachers and are frustrated by the lack of interpersonal contact with the university/college [10]. In addition, some clinical field instructors do not hold the qualifications they are supervising their students to obtain and the university involvement in preparing clinical-field instructors is scant [9]. These obstacles can result in difficulties in communicating about the student’s skill and knowledge development.

Already before the pandemic took hold, tensions existed about expectations to clinical placement capacity from newly developed nursing programs, and the high numbers of students from diverse educational and socioeconomic backgrounds entering these programs [11]. The COVID-19 pandemic put the health and care workforce under unprecedented pressure. The juxtaposition between healthcare having the capacity in providing services to meet the demands of patients is a struggle that can be observed in all countries. Due to the anticipated reduction in nursing workforce following the COVID-19 pandemic, universities and university colleges were instructed by Norway’s authorities to continue delivering nursing education despite knowing there would be problems with providing clinical placements sites and available clinical-field instructors. The Norwegian government passed a temporal instruction on the enactment of education during COVID-19 (Educational instruction covid-19, 2020, §1–3). This instruction provided universities and university colleges flexibility.

Consequently, digital resources for video conferences and lectures, such as Zoom©, Teams©, and Skype©, were introduced to monitor students in clinical placements. This digital transition from face-to face meetings to use of digital resources, was implemented over a short time span. Apart from the clinical placement, which required physical attendance, the nursing students’ learning environment shifted to mainly digital. Hence, the preparatory lectures and student-teacher meetings leading up to the clinical placement were digitally provided. Knowledge about how this process was experienced, can contribute to the development of pedagogical quality in digitalized nursing education.

### Aim and research questions

This study aimed to attain knowledge about university/college teachers’, students’, and clinical-field instructors’ experiences regarding the digital transition in nursing education. The following research questions were developed:

1. How did university/college teachers, students and clinical-field instructors experience the digital transition from face-to- face to digital meetings in the follow-up of students in clinical practice?
2. What were the challenges and benefits of digital evaluation meetings during clinical placements?
3. How was the digital learning environment experienced by university/college teachers, clinical field instructors and students during the ad hoc digitalization?

## Methods

This study used a qualitative exploratory design and applied individual interviews for data collection. Individual interviews are useful when investigating personal experiences and understandings [12]. The interviews were conducted using the digital platforms *Zoom©*. Although face-to-face interviews had been the norm in qualitative interviewing, video-based interviews are proving to also render high-quality data [13]. The interview guide was developed through collaborative discussions by the research team based on earlier practice experiences and literature review. The interview questions addressed expectations and experiences with use of the digital platform, meetings during the clinical placement, benefits and concerns connected to clinical placements and digital meetings and changes in the learning environment. Hence, the interview method was semi-structured interviews. All this study’s authors performed interviews. The interviewers (all women) were nurses and teachers at the selected university/university colleges.

### Participants

We aimed to include participants with a broad range of experiences, consequently recruitments were made from three different educational institutions in Norway, (two Universities, and one University College) representing three nursing education programs and three different graduate levels. The respective educational institutions were in both urban and rural environment and clinical placements were from both primary and tertiary care. Participants comprised three groups: students, clinical-field instructors, and teachers. All participants had either finished their clinical placement (students), supervised students during clinical placement periods in primary care and hospitals (clinical-field instructors) or assessed them (teachers) in the spring of 2020 during the first period of the society lockdown following the onset of the COVID-19 pandemic.

### Recruitment

After the leaders approved of the study, all groups of participants were invited by email by the authors with information about the study and the consent-form. Those who replied positively to the email were invited to a digital interview. In total, 60 invitations were sent to potential participants by mail.

### Setting

The educational institutions comply with the European Credit Transfer and Accumulation System where 60 European credits are the equivalent of a full year of study or work [14]. The three university/university college sites were as follows:

1. A large, urban university. Participants were recruited from a master’s program in advanced geriatric nursing, a program comprising 120 European credits.
2. A university somewhat smaller than Site 1 but in an urban location. These study participants were connected to or attended a mixed master’s/continuing education program 90 or 120 European credits (depending on whether they employed a master’s program or continuing education).
3. A university college significantly smaller than the other two Sites and with a more distant/rural location. These study participants attended a bachelor’s program in nursing comprising 180 European credits.

All three sites held multiple individual evaluation meetings during the clinical placements. An evaluation meeting is a formal session where the student the university/college teacher and the clinical field instructor participate. These meetings included formative evaluations, which are evaluations *for* learning; they reveal what (and how) students are learning. The evaluation forms used in the evaluations were developed by the individual educational institution for each clinical placement based on the learning outcomes for that particular period of clinical placement and adjusted to the specific graduate level. These evaluations aimed to provide the students and clinical-field instructors with an understanding of the students’ performance levels and enable the instructor to adjust accordingly to meet each nursing student’s additional learning needs. There is also an element of assessment or formative evaluation since the students need to pass the clinical placement.

### Analysis

The interviews lasted between 13 and 65 minutes and were transcribed verbatim, resulting in 103 written pages. The analysis was based on Braun and Clarke [15] approach to thematic analysis. Thematic analysis is a method used to identify and analyze themes within a dataset. It consists of six phases: (1) transcribing, reading, and re-reading the data to familiarize oneself with it, while generating codes for the dataset and organizing data relevant to each potential theme; (2) generating codes and (3) searching for themes; (4) reviewing the identifying themes derived from the data; (5) defining and naming themes; and (6) producing the report. All co-authors individually participated in the coding, reviewing, and naming the preliminary themes. To ensure rigor, consensus meetings were held to establish inter-coder agreement in number and naming of themes and sub-themes. The inductive analysis was driven by the research questions and analysis resulted in three main themes and corresponding subthemes addressing all three research questions respectively.

The final analysis is presented in Table 1. The quotes are presented with the corresponding number of participants (ID: 1–19, with prefix ‘S’ for student, ‘I’ for instructor, and ‘T’ for teacher (i.e., “S-2” equals participant number 2, who is a student). The terms university/college teachers, students, and clinical-field instructors refer to bachelor’s, master’s, and continued education levels. The interviewees’ quotes in Norwegian were translated into English by the first author and cross-checked by the coauthors and a language consultant.

**Table 1:** Overview of main themes and sub-themes.

Main themes	Sub themes
*Efficiency compromising pedagogical quality*	***a) Saving time and being on time*** ***b) A challenging learning environment with fluctuating pedagogical quality*** ***c) Concerns about the digital follow-ups of low-performing students*** ***d) Unclear responsibilities regarding digital meetings during clinical placements***
*Digital alienation*.	***a) Unnatural and awkward digital meetings*** ***b) Favoring physical meetings over digital ones*, *especially to establish relationships*** ***c) Feeling lonely and isolated***

### Ethics

Approval to perform this study was granted by the educational leaders of each institution and the Norwegian Center for Research Data (Reference number 557602). All participants received information about the study and gave their written informed consent. There was no potential bias or conflict between researchers and participants, as none of the co-authors had regular close collaboration or supervision of the participants during the project/data collection period.

## Results

Undergraduate- and graduate-level nursing students (n = 5), clinical-field instructors (n = 4), and teachers (n = 9) participated. They were connected to three universities/university colleges representing three nursing education programs in Norway (n = 18). The participants were all women and between 25 and 58 years. The students were the youngest and teachers the oldest.

All participant groups had experienced digital evaluation meetings during the clinical placements. In addition, the teachers and the students also had held or participated in digitally hosted lectures or group reflections in preparation for their clinical placements. They shared stories about their experiences of using both digital contexts. Digital meetings about students’ accomplishments and evaluations of their clinical practice performance took place with the student and clinical-field instructor sitting together, sharing one digital device at the clinical placement site, while the teachers participated digitally from their home/office. The digitally hosted reflection groups and lectures were arranged with the students and the teachers sitting alone in their homes/offices. The most used digital platforms were *Zoom*, *Teams*, and occasionally *Skype*, but telephone and facetime were also used. Web-based learning management and evaluation systems were also used during the students’ clinical placements to provide general information, curriculums, assignments, and evaluation forms.

Two main and seven sub themes regarding the transition to digitized learning environment were identified across all participant groups and presented in Table 1. Main themes were: (1) saving time and increasing efficiency but losing reflection and pedagogical quality; (2) digital alienation and lack of real-life socialization.

### Efficiency compromising pedagogical quality

#### Saving time and being on time

University/college teachers perceived the digital meetings as timesaving and efficient because they did not have to meet at the hospitals/homecare facilities to host physical meetings with the students and clinical-field instructors.

*“All the interrupters were gone*, *and the unnecessary talk disappeared*. *The meetings took shorter time*. *Some of this is positive “(T9)*.

Additionally, the teachers encountered that the clinical-field instructors felt more obligated to turn up at the scheduled meeting time, and thus saved time for all as no one had to wait for the clinical-field instructors to finish a task.

*“The positive experience I had with this*, *is that everybody has actually turned up at the right time”(T17)*.

The students did not mention the meetings’ timesaving element, but some clinical-field instructors found the digital meetings to be more stressful to combine with their clinical nursing work. They thought it was difficult to be on time for the scheduled meetings and that responding to patients’ needs took precedence over the meetings. However, it was more difficult to let the university/college teachers know when they were behind. They found also that the meetings were more efficient, since the time for small talk was reduced and the meetings started right on time.

Students, like the teachers, found the digital reflection groups and digital preparation lecturers both timesaving and flexible.

*“You save a lot of time when you don’t have to travel back and forth*. *And also*, *you can be available at any time”(S2)*.

A few participants did question if the digital platform were actually timesaving since they experienced technical problems with it.

*“You spend a lot of time and effort getting the video call started*, *and you are interrupted by technical challenges and the constant need to check if they can still hear me” (I6)*.

#### A challenging learning environment with fluctuating pedagogical quality

All participants mentioned frustration and wasted time on technical problems while logging in, switching the camera off and on, and a problem with microphones.

*“I know that many students muted or exited the Zoom-room due to technical difficulties”(S10)*.

Students reported missing lectures because of a bad internet connection or low digital competence. In addition, there were different ad hoc solutions for teaching:

“*Some teachers were live on Zoom and had a PowerPoint; some just sent us the PowerPoint with no comments*, *and some sent us the PowerPoint with a recorded audio sound*. *It was not very good quality”(S2)*.

Some university/college teachers said it could even be more time-consuming to give lectures digitally, since they felt that they could not manage the lectures alone. They felt they had to be two teachers present when teaching to manage both the technically and the communication with the students. This combination could make all types of digital meetings more time-consuming.

Most teachers and some clinical-field instructors found the new digital and technology platforms challenging regarding the quality of the pedagogical methods and the communication. The premises for communication, and hence the pedagogical quality, seemed limited by how social interactions were formed by the digital arena:

*“What I don’t get (with the students) is the pedagogical dialogue in a way*, *being together and discussing*,*”(T1)* a teacher said.

For the clinical-field instructors, it was difficult to find time to meet with the teachers and the students. Clinical practice was busier than usual; and sometimes the reflections and informal dimensions of the evaluation meetings were omitted, although priority was given to the assessment dimension.

Some teachers were concerned that the variation, in pedagogical methods, was too narrow and thought it was difficult to use their familiar methods on the digital platform. Students, on the other hand, commented on the lack of a structured plan for digital meetings and lecturers. Lack of an adjusted pedagogy seemed to lower students’ learning.

For the digital preparations and reflections on clinical placements, one teacher advocated that it was easier for students to ask questions during digital lectures and group meetings. One teacher said:

*“We lost some of the reflection in the groups that used to be quite open to each other*. *Because this is the students time to reflect over (their practice)” (T3)*.

The students, however, felt that posing their reflections or asking questions had become more challenging. They increasingly doubted that their thoughts or reflections were important enough. They felt that the spotlight and attention put on them when speaking was challenging, and they felt that they put themselves more “out there.” It was challenging to find the confidence to speak up and express oneself. The students felt it was difficult to motivate and discipline themselves to pay attention during long digital meetings or lectures, especially meetings with low structure and few pauses. It was tiresome to have the camera switched on, but it was too easy to lose concentration when the camera was off.

#### Concerns about the digital follow-up of low-performing students

The teachers and the clinical-field instructors shared extra concern for follow-ups with low-performing students.

*“If there are challenges concerning a student*, *if you are uncertain if the student is in risk of failing*, *it would have been much easier to start that conversation face to face” (I19)*.

They felt it was difficult to handle the process of students at risk of failing the clinical placement. This pertained to the clinical-field instructors, both to the process of informing the students and to not knowing if the teachers shared their opinions. Before, they would have mentioned this informally before meeting with the student; however, with digital meetings only, this had to be discussed and addressed to the student without warning. With a fragile relationship between clinical-field instructors and teachers, this task was demanding for both teachers and students to perform in a coordinated and emphatic way.

*“It is unfortunate if the teacher claims that there is no risk of the student failing*, *but he/she doesn’t perceive the atmosphere in the room” (I5)*.

#### Unclear responsibilities regarding digital meetings during clinical placements

The educational and healthcare institutions did not have a common platform they both could use and master, resulting in modifications between many platforms and hybrid solutions. This meant that technical support from the institutions was low, and the digital platforms used within hospitals, such as the telehealth platforms designed for digital communication with patients, were not used at all. The firewalls with hospital computers made it impossible to communicate and thus blocked access from the digital platforms, such as *Zoom*, and *Teams* at educational institutions. Usually, the students were given (or took) the responsibility to use their own private devices to communicate with the teacher at the school and invited the teacher to the evaluation meeting. While most clinical-field instructors did not mind, one instructor thought that this gave her less influence in the meetings.

### Digital alienation

#### Unnatural and awkward digital meetings

The research participants compared the digital meetings with the familiar non-digital meetings and concluded that they missed important social information. One teacher said,

*“When the camera is on*, *we kind of see each other’s body language*. *I see if you look interested in what I’m saying*, *for example*, *by leaning forward*. *If they are in a zoom meeting with the camera switched off*, *with no audio*, *there is no nonverbal communication” (S8)*.

However, it seemed like something was missing when the camera was on:

*“There is a difference between having the teacher physically visiting or just meeting on Skype or Teams” (S2)*.

Some teachers stated that digital interactions created distance between the students and the clinical placement sites. This could be related to missing eye contact, other nonverbal signs, and gestures, and knowing which person in the meeting the nonverbal communication was directed to. Many students felt less connected with their co-students and their teachers. They were afraid that their message would be misinterpreted and hence misunderstood. Likewise, teachers were afraid that recording their digital lectures stored on the students’ learning platforms would contain errors. This was exhausting and sometimes resulted in shorter meetings since everybody wished that the meetings were over quickly. However, the new situation also created a feeling of fellowship, as mentioned by both teachers and students. One teacher said,

*“We’re in this together” (T9)*.

The feeling of a “COVID crisis” and not having any other choice than to meet digitally made the groups more understanding and tolerant to technical problems. They also showed understanding when their fellow students or co-workers used telephone or *Zoom* without a camera on.

#### Favoring physical meetings over digital ones, especially to establish relationships

Almost every participant favored in-person over digital meetings, especially when the objective was to establish a relationship whether they were meeting for the first time. Using the digital platform only, all the informants felt it was difficult to get to know one another; it was difficult to express insecurities, difficulties, and worries. The teachers and the clinical-field instructors highlighted this:

*“On Zoom*, *one loses the informal part of supervising together” (I6)*.

To compensate for this, many teachers stressed the importance of taking time for small talk during the first meeting and practicing verbalization of ideas that would otherwise be expressed non-verbally.

#### Feeling lonely and isolated

All participant groups, to some extent, felt more alone and isolated when navigating the new COVID-19 digital context. One student put it like this:

*“When most students switch off their cameras and microphone*, *everything is quiet*, *and I wonder if anyone is actually listening to what I’m saying*. *You’re kind of just speaking to yourself” (S3)*.

Teachers commented that this also affected their everyday working environment, and they missed informal meetings in the hallway, for example, seeking advice on how to invite a digital meeting and learning how to share their screen. They lost their collegial support as described by this teacher:

*“It affects the work environment just to be in separate offices*, *but when we also are in our separate houses*, *it is a whole different matter” (T1)*.

Students missed their co-students and informal chats during lunchtime and in the reading room. Of all the groups, the clinical-field instructors felt less lonely. However, they reported that they felt alone about supervising the students when the teachers did not attend the meeting.

## Discussion

Educational institutions faced several challenges during spring 2020, when COVID-19 caused sudden changes and strict restrictions on clinical placement. This study identified two main themes describing nursing students’, clinical-field instructors’, and university/college teachers’ experiences with the ad hoc digitalization caused by COVID-19 with emphasis on digital communication and education: 1) Saving time and increasing efficiency but losing reflection and pedagogical quality and 2) digital alienation and lack of real-life socialization. The results shows how the ad- hoc digital transformation maintained and amplified the pre- pandemic concerns of quality in nursing education, in particular how to meet the students’ educational needs from all income levels [16]. Sudden changes in education seemed to affect the students’ achievement negatively, especially among low-income and low-achieving students [17]. The results from this study supports earlier research identifying how students were dissatisfied with online learning in general during the pandemic [18] but adds to a subtle understanding of how it was experienced.

### Difficulties in achieving and assessing learning outcomes

A concern raised by the teachers was that some nurses educated during the pandemic could not acquire the same level of skills as previously educated nurses. Similarly, Ulenaers, Grosemans [19] identified how some students experienced less advanced learning situations due to changes in clinical placement sites. Some hospital units stopped all planned hospital attendances other than emergency visits, resulting in fewer patients and less diversity in learning opportunities for some of the students. On the opposite side, in busy COVID-19 units, students were not included in relevant learning situations because the learning environment was too busy. Nursing students’ clinical learning is influenced by the nurses’ workload and the job intensity at their clinical placement [20].The lack of learning opportunities and unsatisfactory skill acquisition were also identified by the students during clinical placement in a study by Kaveh, Charati [21]. The lack of relevant learning situations for some students was worrisome, specifically because of the importance of the clinical training in nursing education [22].

Key results from this study suggest shared concern from both teachers and clinical-field instructors about losing students reflections because of the digital transition with bad internet connections or low digital competence. These challenges potentially also made students absent from lectures and discussions. Low digital competence was worrisome, especially since digital competence was a vital part of digital literacy. Educators’ competencies should also be enhanced [23] and should include strengthening the teachers’ abilities to plan and to conduct ICT-supported teaching and apply current research in supporting students’ learning [4]. Digital literacy is becoming an increasingly important component of clinical practice and nursing education since it is vital in accessing evidence for use in clinical practice [23].

### Challenges in assessing students

Evaluating the students’ learning process during clinical placement became more difficult when the teacher was not physically present. The teachers and clinical-field instructors were especially concerned with low-performing students. Similar concerns have been identified in earlier studies. Black, Curzio [24] pointed to how having to fail an underperforming student can be morally distressing for clinical-field instructors. This process can be even more difficult without support from the teacher at the school. A qualitative study by Hunt et al. [25] showed that clinical-field instructors needed support to feel confident enough to fail underperforming students. Furthermore, learning and evaluating practical skills in nursing education depends on triangulation between the student, instructor, and teacher [26, 27]. When the assessment is digital and the instructor can feel low social support, this can lead to higher thresholds for failing students and less attention being paid to the assessment of the students’ skills. Especially if field instructors are unsure about their own qualifications which Broadbent, Moxham (9) identified in their study. University involvement and close follow up of and with clinical-field instructors seems to be important to obtain a thorough and formative evaluation of the students’ performance during the clinical placement.

### Social alienation

All participants felt that the digital platforms were difficult to use and that it was an unnatural setting for reflection and learning. In addition, both the teachers and the students felt alone and isolated. This is in line with earlier studies identifying how loneliness among students was high during the coronavirus pandemic [28]. Also Brennan [29] who identified how teachers in higher education felt isolated and disconnected due to student reluctance to use webcams and the techno-overload due to the changes in teaching format, from face-to-face to online. This is often referred to as technostress [30]. In addition, Luchetti, Lee [31] pointed to the effects of the pandemic’s stay-at-home orders had on general psychological well-being. Our results showed how social alienation experienced because of the digitalization of clinical placements, aggravated the situation. This can be especially challenging for nursing students’ since the emotional burdens influence their ability to learn during clinical placements [20] and are worsened by lack of structure and predictability [32] This adds to the difficulties created by less adequate places for clinical placements and highlights how difficult the situation was.

The teachers and the clinical-field instructors’ difficulties in assessing the students, the social alienation experienced by both the teachers and the students; and the sometimes ill-prepared, ad hoc lectures and meetings, resulted in a fluctuating pedagogical quality during the start of the COVID-19 pandemic. We questioned if the quality assurance concerning students’ skill levels and evaluations during their clinical placements, may have resulted in a risk of lower-performing newly graduated nurses compared with previously graduates.

### Study limitations

A strength of this study is the multiple perspectives provided by students, clinical-field instructors, and university/college teachers. They revealed that the ad hoc digital transition across different nursing disciplines caused changes and quality challenges during clinical placements. Another strength of this study is the variation in sampling at the organizational and participant levels. The participants came from three educational sites with different sizes and three different degrees in nursing. To enhance the reflexivity with the study, the analysis was conducted by the research team, who based on their different academic standings and institutions from which they came, provided a range of perspectives to the data interpretation.

Most teachers agreed to participate in this study, but a higher number of the students and clinical field instructors did not respond or declined. Consequently, only a small number of students and clinical field instructors participated in this study, which is a limitation. The results must therefore be interpreted with caution. The teachers and clinical-field instructors were included regardless of pedagogical training and/or pedagogical background.

All interviews were performed digitally; therefore, our digitally hosted conversations with the participants may have the same problems as those described by the participants in this study, namely, a potentially awkward interview situation.

## Conclusion

The COVID-19 pandemic resulted in ad hoc solutions for clinical placements, assessments, and evaluations. Some students and teachers experienced a challenging learning environment. Because of this, the teachers and clinical-field instructors were concerned about the consequences such as potential for lower-performing students in this group compared to the previous graduated nursing students. In this study we explored one aspect of the digital transformation, the role of online assessments and evaluations in clinical placement. In line with our results, more knowledge about the student-instructor relationship, pedagogy, the role of the instructor, as well as the role of the student, are needed. Whether this potential disadvantage experienced by these students during their clinical placements will need to be rectified later in their education, needs to be further explored. The results from this study have the potential to inform nursing education about how to prepare for and to manage use of ICT in nursing education in the future.

## Supporting information

S1 Appendix Interview guide. (DOCX)
