# Supplementary material for: Ad hoc digital communication and assessment during clinical placements in nursing education; a qualitative research study of students’, clinical instructors’, and teachers’ experiences
Source: PLoS One. 2023 Jul 21;18(7):e0287438. doi: 10.1371/journal.pone.0287438 (PMC10361501; doi:10.1371/journal.pone.0287438)
Supplement: S1 Appendix — (DOCX) [file pone.0287438.s001.docx]

**Appendix 1 Interview guide**

Ad Hoc Digital Communication and Assessment during Clinical Placements in Nursing Education; A Qualitative Research Study of Students', Clinical Instructors', and Teachers'; experiences

**Interview questions for students**

Have you completed a similar clinical placement before?

Can you describe how you were followed up digitally by the university in this clinical placement?

How did you experience this clinical placement?

What expectations did you have to the college/university teachers during the clinical placement?

How did you experience the contact/collaboration with your college/university teacher?

What digital software was used when communicating with the college/university teacher?

How did the digital platform work for you?

How did your experience the conversations you had with your clinical instructors and the college/university teacher on digital solutions?

If any, what were the challenges or disadvantages with solely using digital follow-up with the college/university teachers?

If any, what were benefits?

If any, what other changes were carried out during this clinical placement as a result of the Covid-19 pandemic?

How have these changes affected this clinical placement period?

Is there anything else you would like to share regarding digital follow-up of students in clinical placement?

**Interview questions for clinical instructors**

Have you followed up with the students in a similar fashion as previous clinical placements before COVID-19?

Can you describe how the students were followed up digitally by the college/university teachers in this clinical placement?

How did you experience this clinical placement?

What expectations did you have with the students and college/university teachers during the clinical placement?

How did you experience the contact/collaboration with the college/university teacher?

What digital software was used when communicating with the college/university teacher?

How has the digital platform worked for you?

How did you experience the conversations you had with the student and the college/university teacher on digital solutions?

If any, what were the challenges or disadvantages with solely using digital follow-up with the students by the college/university teachers?

If any, what were benefits?

If any, what other changes were carried out during this clinical placement as a result of the Covid-19 pandemic?

How have these changes affected how you followed up with the students in this clinical placement period?

Is there anything else you would like to share regarding the digital follow-up of students in clinical placements?

**Interview questions for college/university teachers**

Have you followed up students in a similar fashion as previous clinical placements before COVID-19?

Can you describe how you followed up with students digitally in this clinical placement?

What expectations did you have with the students and the clinical instructors during the clinical placement?

How did you experience the contact/collaboration with the students and clinical instructors?

What digital software was used when communicating with students and clinical instructors?

How did the digital platform work for you?

What did you experience during the conversations you had with the students and the clinical instructors on digital solutions?

If any, what were the challenges or disadvantages with solely using digital follow-up with the students in a clinical placement?

If any, what were benefits?

If any, what other changes were carried out during this clinical placement as a result of the Covid-19 pandemic?

How did these changes affect how you followed up the students during this clinical placement period?

Is there anything else you would like to share regarding the digital follow-up of students in clinical placement?
